# Effects of cell-free DNA on kidney disease and intervention strategies

**DOI:** 10.3389/fphar.2024.1377874

**Published:** 2024-05-21

**Authors:** Mingying Zhang, Yubin Cai, Xiaoze Zhong, Weijun Liu, Yuan Lin, Zhanyi Qiu, Ruihuang Liang, Huibo Wei, Kefei Wu, Qinghua Liu

**Affiliations:** ^1^ Department of Nephrology, Jieyang People’s Hospital, Jieyang, China; ^2^ Department of Nephrology, The First Affiliated Hospital, Sun Yat-sen University, Guangzhou, China; ^3^ NHC Key Laboratory of Clinical Nephrology (Sun Yat-sen University) and Guangdong Provincial Key Laboratory of Nephrology, Guangzhou, China

**Keywords:** acute kidney injury, chronic kidney disease, cell-free DNA, neutrophil extracellular traps, nanomaterial

## Abstract

Kidney disease has become a global public health problem. Patients with end-stage kidney disease must rely on dialysis or undergo renal transplantation, placing heavy burdens on their families and society. Therefore, it is important to develop new therapeutic targets and intervention strategies during early stages of chronic kidney disease. The widespread application of liquid biopsy has led to an increasing number of studies concerning the roles of cell-free DNA (cfDNA) in kidney disease. In this review, we summarize relevant studies concerning the roles of cfDNA in kidney disease and describe various strategies for targeted removal of cfDNA, with the goal of establishing novel therapeutic approaches for kidney disease.

## 1 Introduction

The kidneys are vital organs for urine production, acid-base balance, and the maintenance of endocrine function (Dhondup and Qian, 2017). Renal impairment may lead to hypertension, anemia, heart failure, pericarditis, cardiomyopathy, electrolyte disturbances, acid-base imbalances, renal osteopathy, infection, and multiple serious complications (Dhondup and Qian, 2017). Renal insufficiency is mainly caused by acute kidney injury (AKI) or chronic kidney disease (CKD). AKI is a clinical syndrome that comprises a sudden (within 1–7 days) and sustained (>24 h) decline in renal function (Kellum et al., 2021). Previously, the long-term prognosis was considered satisfactory in patients whose renal function recovered after AKI. However, since 2008, there has been increasing evidence of a strong association between AKI (including mild cases) and the subsequent development of CKD. Studies consistently showed that a large number of patients tend to partially recover after AKI; however, regardless of underlying kidney disease status, such patients gradually progressed to CKD and end-stage kidney disease (Chawla et al., 2014). In 2013, a cross-sectional study in China revealed that the incidence and mortality of AKI in hospitalized patients were approximately 0.99%–2.03% and 12.8%, respectively. Moreover, the length of stay and in-hospital mortality rate were both positively correlated with AKI stage, and 50% of patients with AKI eventually progressed to CKD (Yang et al., 2015). The international multi-center AKI-EPI study showed that more than 50% of patients in intensive care units had AKI; thus, AKI is considered a global public health problem. In 2020, the coronavirus disease 2019 (COVID-19) pandemic spread worldwide; one-third of hospitalized patients with COVID-19 developed AKI (Legrand et al., 2021). In the United States, 46% of hospitalized patients with COVID-19 developed AKI; 19% of these patients required dialysis, and the short-term mortality rate approached 50%. In 35% of patients, renal function did not recover to baseline status after discharge (Chan et al., 2021). Patients with COVID-19 typically exhibited comorbid AKI; mortality was much higher among such patients than among patients with COVID-19 who did not develop AKI. Therefore, the long-term prognosis of patients with COVID-19 who have AKI is likely to become a global public health problem (Gaudry et al., 2019; Liu et al., 2020). The specific mechanism of COVID-19-related AKI is unclear. Although it could be attributed to ischemia–reperfusion injury (IRI) and infection (as previously reported), it is more often associated with local inflammation secondary to systemic inflammatory storm, endothelial injury, and renal microthrombus formation (Kellum et al., 2021). Thus, in addition to conventional fluid resuscitation and anti-infection therapies, anti-inflammatory drugs such as steroids and interleukin (IL)-6 receptor blockers may prevent AKI progression (Legrand et al., 2021).

Compared with AKI, CKD is more insidious. This disease comprises a class of chronic renal structural and functional disorders (i.e., sustained renal damage for >3 months) that gradually lead to permanent renal damage through various mechanisms; they eventually progress to end-stage kidney disease requiring dialysis or renal transplantation with high life-support costs. CKD is also an important risk factor for AKI, such that long-term mortality or end-stage kidney disease risk increases by 30% among CKD patients with concomitant AKI (He et al., 2017; Hapca et al., 2021). The global prevalence of CKD is rapidly increasing. In 2017, the global prevalence of CKD was 9.1% (∼697.5 million patients), and 12 million deaths from CKD had been reported. Almost one-third of CKD patients were from China (130 million) and India (120 million) (Collaboration, 2020). In China, the prevalence of CKD is approximately 10.8%; it is expected to worsen in the next 10 years, possibly because of rapid increases in morbidity (e.g., diabetes, hypertension, and obesity) and other CKD risk factors (Wang et al., 2023). For various reasons, most patients exhibit end-stage kidney disease at the time of diagnosis. Resources for early diagnosis and management of AKI and CKD are insufficient, and treatment options to prevent or delay kidney disease are limited (Zhang et al., 2012; Lin et al., 2014). Current AKI treatment is primarily based on conservative measures (e.g., drug-mediated management of hyperkalemia and fluid overload) and timely renal replacement therapy (Gaudry et al., 2019). Drug therapies for CKD mainly include steroids and immunosuppressants, which can cause systemic adverse reactions because of their non-targeted extrarenal distribution dynamics (Liu et al., 2019). Even within the kidney, these drugs may not act on specific targeted cells, especially in pathological conditions such as tubulointerstitial fibrosis, glomerulosclerosis, and fibrosis-induced reduction of vascular beds (Zhou et al., 2014). Therefore, it is important to explore new therapeutic targets in kidney disease.

The results of recent studies have suggested that small free circulating DNA fragments (i.e., cell-free DNA [cfDNA]) are present in the peripheral blood of healthy individuals and various types of patients; this free and partially degraded endogenous DNA is located outside of cells. Extracellular cfDNA is produced from necrotic or apoptotic cells, providing a continuous measure of cell death *in vivo* (Wan et al., 2017). This discovery led to the emergence of liquid biopsy, a new diagnostic modality with applications in multiple medical fields. Examples include non-invasive prenatal testing; screening for fetal genetic defects in high-risk pregnancies (Fan et al., 2012); early recognition of post-transplant rejection (De Vlaminck et al., 2015); and assessments of circulating mutated DNA in cancer detection, typing, and surveillance (Wan et al., 2017). In addition to these clinical applications, increasing numbers of diseases have been associated with cfDNA (García-Pardo et al., 2022). In this review, we summarize cfDNA sources, distributions, contents, and detection methods; we also discuss the roles of cfDNA in the pathogenesis of AKI and CKD, along with mechanisms of tissue damage. Finally, we explain strategies for targeted removal of cfDNA from the kidneys, with the goal of establishing novel therapeutic approaches for kidney disease.

## 2 cfDNA sources, distribution, content, and detection methods

### 2.1 Sources of cfDNA

Because cell-derived degraded DNA fragments are free in extracellular space, they can be regarded as uncoated DNA (Azad et al., 2015). cfDNA consists of cell-free mitochondrial DNA (cf-mtDNA) and cell-free nuclear DNA (cf-nDNA) (Chang et al., 2019).

There are four main biological sources of cfDNA. The first source is any form of cell death, such as apoptosis or necrosis, pyroptosis, autophagy, phagocytosis and NETosis (Grabuschnig et al., 2020). Chromatin is almost completely degraded during apoptosis or necrosis; naked DNA fragments can be degraded by intracellular nucleases, and intact protein-bound DNA fragments can be released into the circulation because of their protein envelopes (Mondelo-Macia et al., 2021). The second source is graft or fetal free DNA. Fetal DNA is released into maternal blood, allowing Y chromosome detection in pregnancies with a male fetus. cfDNA can also be used for non-invasive prenatal Down syndrome screening (Vrachnis et al., 2014; Malmir et al., 2021) and immune rejection surveillance after organ transplantation (Snyder et al., 2016; Danovitch et al., 2021). The third source is the release of auto-activated DNA, cfDNA content is related to trauma, burn, infection, heart failure, stroke, and organ IRI level (Tian et al., 2019; Dinsdale et al., 2020; Yokokawa et al., 2020; Zuo et al., 2020; Saravanan et al., 2021). The fourth source is tumor-derived ctDNA, which comprises a very small proportion of cfDNA. However, ctDNA testing is widely throughout tumor diagnosis and treatment: from early diagnosis and screening of solid and hematological tumors to assessments of dynamic changes in ctDNA content according to treatment response, as well as enrollment in interventional clinical trials based on ctDNA content (Cescon et al., 2020).

Because pathophysiological processes, pathogenic mechanisms, and tissue microenvironments differ among diseases, cfDNA production involves a complex process. No single mechanism can fully explain the source of all cfDNA; typically, two or more production mechanisms are involved (García-Pardo et al., 2022).

### 2.2 cfDNA fragment distribution

The peak of the cfDNA fragment length distributed at around 166 bp (corresponding to chromosome length), and has 10.4 bp of periodic fluctuations in the range of 100–160 bp, corresponding to the pitch of nucleosome core DNA. The cfDNA fragment distribution is related to nucleosome structure. Each nucleosome unit consists of one histone octamer, one DNA molecule, and one histone H1 molecule. A nucleosome core particle is formed from the histone octamer, which is supercoiled in 1.75 circles by a 146-bp DNA molecule; histone H1 binds an additional 20-bp DNA molecule outside of the core particle to inhibit further entry and exit of nucleosome DNA, stabilizing the nucleosome. Therefore, the cfDNA fragment distribution characteristics are clear: the junction DNA between adjacent nucleosomes (i.e., naked DNA) is degraded by nucleases, whereas DNA wrapped around the nucleosome core particle is protected. The 146-bp DNA molecule wrapped around the core particle and the 20-bp DNA molecule bound to histone H1 collectively correspond to the main peak of 166 bp; these DNA molecules undergo cleavage or lysis in a manner influenced by DNA spacing, leading to smaller peaks across the range of 100–160 bp (Snyder et al., 2016).

### 2.3 cfDNA content

In healthy individuals, plasma cfDNA is assumed to originate from normal hematopoietic cells, such as leukocytes (55%), red blood cell progenitors (30%), vascular endothelial cells (10%), and hepatocytes (1%). Generally, the cfDNA content is low in healthy individuals; plasma cfDNA is mostly protein-bound and exists in complexes, with relatively few dissociated DNA fragments. cfDNA is present in both plasma and serum fractions of blood, and the cfDNA content in the peripheral blood of healthy individuals is typically ∼100 ng/mL. However, in patients with cancer, the cfDNA content can reach 1,000 ng/mL. Elevated cfDNA content has been observed in various diseases, such as infection, trauma, postoperative conditions, parasitic diseases, cardiovascular diseases, kidney diseases, and hematological disorders (Moss et al., 2018).

### 2.4 cfDNA detection methods

Since the emergence of precision medicine, targeted drugs and immunotherapy have become viable strategies in various medical fields. Moreover, liquid biopsy has become an important diagnostic modality with non-invasive, sensitive, and dynamic properties. In recent years, liquid biopsy based on accurate detection of cfDNA by the fluorescent dye PicoGreen and nucleic acid aptamers has been used for non-invasive diagnostic and prognostic analyses of multiple diseases. Blood, urine, feces, cerebrospinal fluid, saliva, pleural fluid, and ascites can all be used as cfDNA detection samples (Aucamp et al., 2018).

## 3 cfDNA and kidney disease

### 3.1 cfDNA and AKI

Recently, there has been increasing evidence that the cfDNA content can be used as a predictor of AKI; for example, it can serve as a marker of clinical outcomes in patients with sepsis (Jing et al., 2022). In patients with sepsis and comorbid AKI, especially patients requiring continuous renal replacement therapy, an increased cfDNA content is associated with poor prognosis. Levels of cysteine protease-3 (caspase-3), IL-6, and IL-18 are higher in patients with sepsis; these increased levels are associated with cfDNA content (Clementi et al., 2016). Furthermore, in patients undergoing cardiac surgery with cardiopulmonary bypass, a high plasma cfDNA content can accurately predict the occurrence of late postoperative AKI. Activated neutrophils trigger systemic inflammation, leading to the release of neutrophil extracellular traps (NETs). Nuclear DNA released from necrotic and apoptotic cells may contribute to an increase in cfDNA content. cfDNA and NETs can cause endothelial damage and organ dysfunction. On postoperative day 3, compared with neutrophil gelatinase-associated lipocalin (area under the curve [AUC] = 0.699) and creatinine (AUC = 0.688), cfDNA was the best predictor of AKI (AUC = 0.804) at a threshold of 260.53 ng/mL. With a sensitivity of 87.5% and specificity of 64.9% and a highest diagnostic odds ratio of 13.1 when compared with creatinine, a traditional value used in clinical practice. Surveillance of cfDNA content beginning on the first postoperative day may be a valuable tool for predicting late AKI after cardiac surgery without cardiopulmonary bypass (Merkle et al., 2019). All of the above studies demonstrated that cfDNA content can serve as a predictor of AKI.

**FIGURE 1:**
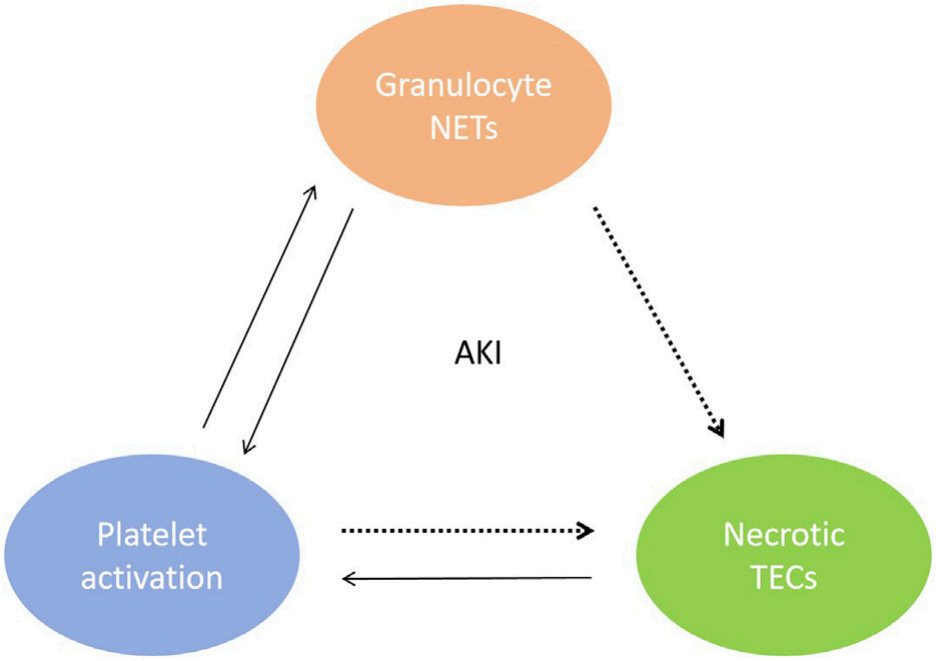
Mutual malignant triad between renal tubular epithelial cell necrosis, activated platelets and NETs during AKI. cfDNA from necrotic TECs elicits platelet activation, interactions between platelets and granulocytes promote the formation of NETs.

*In vitro* and *in vivo* studies have elucidated the pathological mechanisms underlying the presence of cfDNA in AKI. During periods of renal IRI, cfDNA from necrotic renal tubular epithelial cells (TECs) is released into the tubulointerstitium and blood circulation, activating NETs through various pathways. First, cfDNA elicits platelet activation; interactions between platelets and granulocytes promote the formation of NETs (Jansen et al., 2017), leading to further exacerbation of kidney inflammation and tissue damage (Gupta and Kaplan, 2016; Liu and Dong, 2018). As shown in Figure 1. Second, cfDNA-mediated stimulation of Toll-like receptors (TLRs) on the neutrophil cell membrane causes calcium ion (Ca^2+^) release from the endoplasmic reticulum. This process leads to protein kinase C activation and assembly of the nicotinamide adenine dinucleotide phosphate (NADPH) oxidase complex, which promotes mitochondrial activation and subsequent reactive oxygen species (ROS) production. Finally, the nuclear membrane is degraded, and a mixture of chromatin and granular proteins is ejected from the cell to form NETs (Gupta and Kaplan, 2016). Third, renal tubule necrosis and NETs formation trigger distal organ dysfunction by releasing cytokines and histones (Nakazawa et al., 2017). NETs also cause renal vasculature occlusion promote vaso-thrombosis that aggravates AKI (Xu et al., 2024). In 2018, Liu et al. published a review article emphasizing that cfDNA-mediated activation of NETs can serve as a new therapeutic target for AKI (Liu and Dong, 2018).

Additional studies in recent years have shown that cf-mtDNA contents in blood and urine are correlated with AKI. Whitaker et al. found that the urine cf-mtDNA content was closely associated with ischemia duration and AKI progression after cardiac surgery (Whitaker et al., 2015). Similarly, Hu et al. demonstrated a robust correlation between urinary cf-mtDNA and AKI severity in patients with sepsis and surgery-related critical illness (Hu et al., 2017; Hu et al., 2018). In their study, the urinary cf-mtDNA content was significantly inversely correlated with renal mtDNA content, adenosine triphosphate (ATP) content, and the expression of mitochondrial genes such as PGC-1α and NDUFB815, 24, suggesting that mitochondrial dysfunction is involved in AKI (Whitaker et al., 2015; Hu et al., 2017). Other transplant-related studies have shown that increased extracellular cf-mtDNA content is associated with increased inflammatory cytokine levels and delayed graft function after kidney transplantation (Jansen et al., 2020). These findings indicated that cf-mtDNA could act as an important IRI suppressor after kidney transplantation. Renal IRI is the leading cause of AKI and post-transplant allograft dysfunction; it causes ROS production, microvascular damage, inflammation, and cell death, resulting in allograft failure (Ponticelli, 2014).

The mitochondrial damage associated with IRI can cause AKI progression through several pathophysiological processes, including mitochondrial permeability switch pore opening, ROS release, ATP depletion, and the release of mitochondrial damage-associated molecular patterns, including N-formyl peptide, cytochrome c, and cf-mtDNA. Decreased mitochondrial gene expression and increased ATP depletion are also associated with altered mitochondrial homeostasis and the impairment of kidney repair processes after AKI (Jassem and Heaton, 2004; Emma et al., 2016). An weakened mitochondrial antioxidant defense system increases ROS production, resulting in mtDNA damage (Bhargava and Schnellmann, 2017). Thus, mtDNA fragments are released from mitochondria via mitochondrial permeability switch pores or necrosis (Patrushev et al., 2004; Kaczmarek et al., 2013).

The above studies illustrated the origin of cf-mtDNA during AKI. However, when Fang et al. explored pathogenic mechanisms involving cf-mtDNA, they found that cf-mtDNA could activate TLR9 and NLR family pyrin domain containing 3 (NLRP3) to mediate inflammatory responses (Shimada et al., 2012; Fang et al., 2016). The inhibition of cf-mtDNA release could suppress the formation of inflammatory responses, suggesting that there is positive feedback between cf-mtDNA and NLRP3-mediated inflammatory responses (Nakahira et al., 2011). Tsuji et al. demonstrated that cf-mtDNA stimulates cytokine production and mitochondrial damage in the kidney through a TLR9-related pathway (Tsuji et al., 2016), but further investigation is needed to elucidate the detailed mechanism.

### 3.2 cfDNA and CKD

A comparison of cfDNA content between CKD patients and healthy individuals indicated that cfDNA content did not significantly differ between groups. Further comparison of changes in plasma cfDNA content according to peritoneal dialysis and hemodialysis statuses showed that cfDNA content was higher in CKD patients undergoing hemodialysis than in CKD patients who did not require dialysis. Among patients undergoing peritoneal dialysis, the cfDNA content in overnight peritoneal dialysis fluid was inversely proportional to the duration of peritoneal dialysis therapy. These results suggested that the kidneys are not the main organs for removal of circulating cfDNA (Korabecna et al., 2008). However, in 2019, Watson et al. found that levels of urinary cfDNA and methylated cfDNA combined with other KIT score indicators could predict various stages of CKD; thus, cfDNA may be a superior indicator of early CKD, compared with conventional indices such as creatinine (Watson et al., 2019). In 2019, Chang et al. found that plasma cf-nDNA content was significantly associated with disease severity in CKD patients; notably, plasma cf-nDNA content was significantly lower in patients with advanced CKD. Low levels of urinary cf-mtDNA and cf-nDNA were significantly associated with a good renal outcome at the 6-month follow-up. Thus, baseline levels of urinary cf-mtDNA and cf-nDNA could predict the renal outcome at 6 months (Chang et al., 2019). Discrepancies in the above research results may be related to factors such as sample size and detection method. Therefore, cfDNA-related changes and mechanisms of action in CKD patients have not been fully elucidated.

Considering the persistent mitochondrial damage after AKI (Lan et al., 2016; Szeto et al., 2017), it is important to determine whether there is a link to CKD onset. In 2016, Tin et al. published a cohort study of 1,490 participants with a mean follow-up duration of 19.6 years; they found that a higher mtDNA copy number in blood corresponded with a lower risk of CKD. This association remained statistically significant after adjustments for other CKD risk factors, including diabetes mellitus, hypertension, C-reactive protein level, and white blood cell count. These results suggested that further analyses of factors affecting the mtDNA copy number in blood could facilitate CKD prevention and treatment, based on the conclusion that the functional status of mtDNA in blood is associated with CKD prognosis (Tin et al., 2016). Two other studies showed that the urinary cf-mtDNA content was associated with renal function and kidney scarring in CKD patients (Wei PZ. et al., 2018; Wei Z. et al., 2018). Szeto et al. revealed that mitochondrial injury in podocytes and proximal renal tubular cells persisted for 9 months after renal ischemic injury, leading to inflammation and CKD (Szeto et al., 2017). Previous studies also showed that mitochondrial biological function was essential for kidney repair; persistent mitochondrial dysfunction led to tubular epithelial cell–interstitial transformation and renal fibrosis (Yuan et al., 2012). The above studies demonstrated that mtDNA content was correlated with renal injury and fibrosis progression in CKD.

Autoimmune disease-mediated renal damage is also an important cause of CKD; common causes include systemic lupus erythematosus (SLE) and antineutrophil cytoplasmic antibody vasculitis. Recent studies demonstrated that cfDNA content was higher in SLE patients than in healthy controls, and the degree of difference was related to disease activity. These preliminary results indicated that cfDNA content could serve as an indicator of current and future disease activity in SLE patients (Atamaniuk et al., 2011; Xu et al., 2018). SLE can cause chronic inflammation, injury, and cell death in various tissues. Owing to the increased numbers of dying cells, heterozygous mutations of encoding nucleases, low levels of nuclease, and reduced ability of extracellular or intracellular nucleases to degrade cfDNA, increased cfDNA content mediates immune cell activation and promotes inflammatory circulation (Cepika et al., 2012; Elkon, 2018)^.^ In addition to AKI, DNA/NET-mediated tissue damage has been observed lupus mouse models, where DNA/NET-mediated endothelial cell to stromal cell transformation occurred; this process promoted kidney fibrosis (Pieterse et al., 2017). Increased cfDNA content was detected in patients with eosinophilic granulomatous polyangiitis (EGPA); the level of increase was dependent on disease activity. The presence of DNase-resistant eosinophil extracellular traps in small vascular thromboses might lead to increased cfDNA content in EGPA, followed by the formation of EGPA immunothrombosis (Hashimoto et al., 2021).

### 3.3 cfDNA and renal allograft failure

In kidney transplantation, donor-derived cell-free DNA (dd-cfDNA) is a non-invasive transplantation liquid biopsy biomarker with greater sensitivity and specificity than serum creatinine for early accurate detection of acute and chronic rejection after transplantation. The identification of any rejection is crucial for the long-term survival of transplanted kidneys. dd-cfDNA has a low false negative rate in terms of detecting rejection, reducing the need for clinically unnecessary renal biopsy. Furthermore, dd-cfDNA can detect immune activation-related graft damage caused by insufficient immunosuppressant use, aiding in immunosuppressant dosage guidance (Oellerich et al., 2021). Serial monitoring of urinary dd-cfDNA after renal transplantation is sensitive to detect acute injury in the donor organ (Sigdel et al., 2013; Knight et al., 2019; Chen et al., 2022). Elevated urine dd-cfDNA could also used in discriminating BK polyomavirus-associated nephropathy in recipients infected with BK virus (Chen et al., 2020).

## 4 Strategies for cfDNA removal

How cfDNA is cleared from the plasma remains unknown (Celec et al., 2018) and kidney plays minor role in cfDNA clearance (Gauthier et al., 1996; Korabecna et al., 2008). The above-mentioned studies showed that cfDNA from necrotic or apoptotic renal tubular epithelial cells is released into the renal interstitium and blood circulation during AKI, where it mediates NETs formation through the activation of granulocyte TLRs or platelets to promote immune responses and inflammatory reactions that exacerbate renal injury. Furthermore, the weakened mitochondrial antioxidant defense system allowed increased ROS production, leading to mtDNA damage. Next, cf-mtDNA is released from mitochondria, activating TLR9 and NLRP3 to mediate inflammatory responses and renal injury. Although there remain questions concerning changes in the circulating cfDNA content during CKD onset and their effects on CKD, it is clear that a higher mtDNA copy number is associated with a lower risk of CKD. Persistent mitochondrial dysfunction leads to tubular epithelial cell–interstitial transformation and renal fibrosis. Therefore, during renal injury, renal immune response-mediated excessive inflammatory responses and kidney fibrosis may be reduced by inhibiting NETs formation and enhanced ROS production by over-activated mitochondria. These two processes may be achieved through the removal of pathologically excessive DNA produced by renal tubular epithelial cell necrosis or mitochondrial damage (Gupta and Kaplan, 2016; Liu and Dong, 2018), potentially providing novel therapeutic strategies for kidney disease.

**FIGURE 2:**
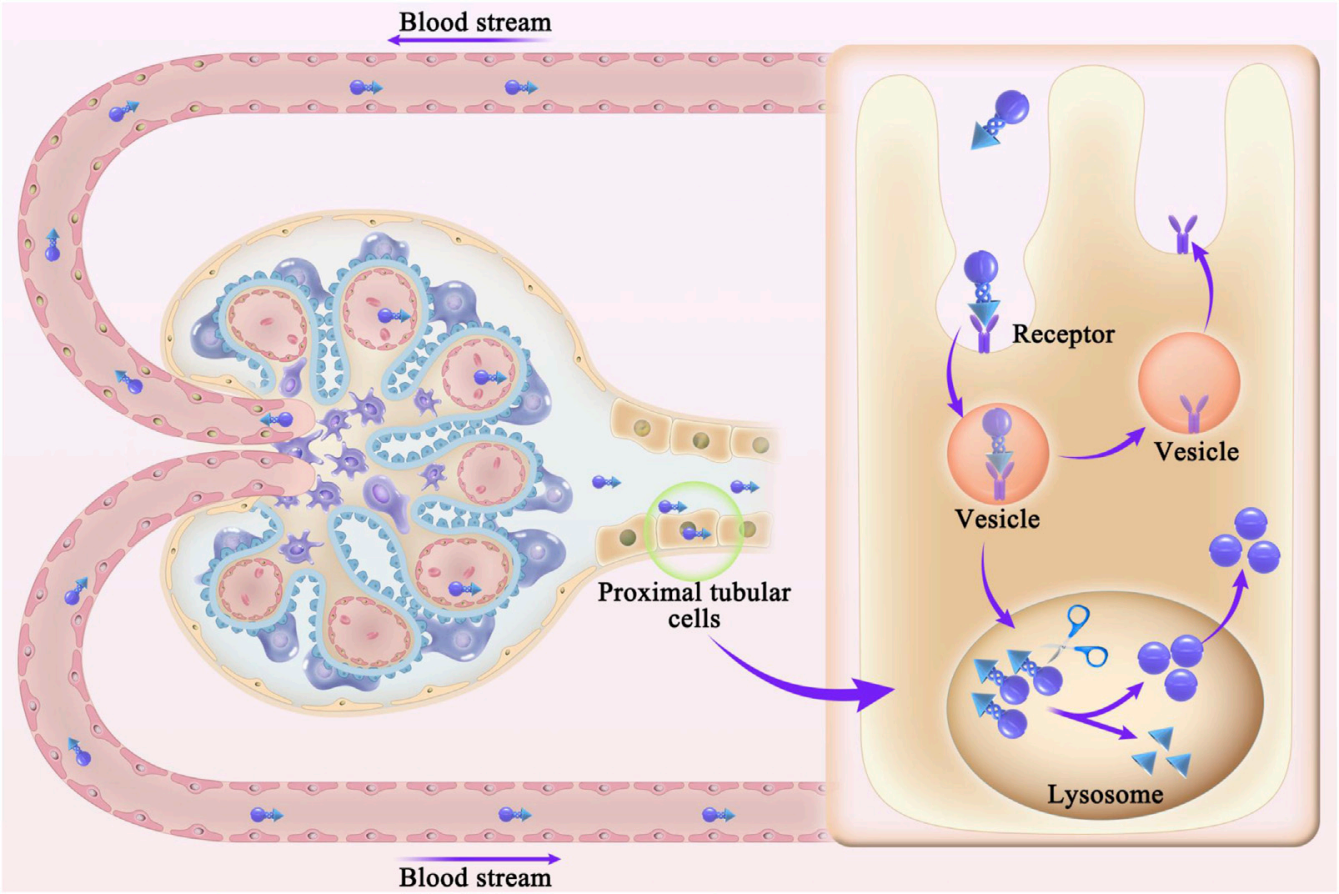
Schematic diagram of nanodrug release and deposition in the glomerular mesangial region. Spaces between glomerular capillary endothelial cells and podocytes are 60–80 nm and 20–50 nm, respectively. Drug delivery system in the range of 70–130 nm (liposomes or nanoparticles) can reach the mesangial region without glomerular filtration and effectively remain in this region.

The development of safe and efficient cfDNA removal strategies is a key clinical focus and major challenge in biomedical research. cfDNA contains various structures, sequences, and modifications; thus, it is difficult to specifically neutralize cfDNA via complementary sequences or structural interactions (Kilpinen et al., 2013). Current treatments for kidney disease are mainly based on the systemic administration of steroids or immunosuppressants. However, systemic administration may not be effective for specific renal target cells, especially in pathological conditions such as renal tubulointerstitial fibrosis, glomerulosclerosis, and decreased renal vascular bed or renal blood flow (Zhou et al., 2014; Liu et al., 2019). Advances in precision medicine and biomedical materials have led to new opportunities for the development of cfDNA removal strategies with organ-targeting therapeutic effects. In 2020, Liang et al. found that cfDNA released from damaged or dead cells could activate DNA sensors, exacerbating the progression of rheumatoid arthritis. Based on this finding, cationic nanoparticles of approximately 40 nm were developed to remove cfDNA, thereby inhibiting the activation of joint synovial fluid monocytes and fibroblast-like synovial cells. In rat models of CpG-induced and collagen-induced rheumatoid arthritis, intravenous cationic nanoparticle administration relieved arthritis symptoms. These results indicated new possibilities for the treatment of inflammatory diseases with cfDNA removal-targeting nanomedicine materials (Liang et al., 2018). However, Dawulieti et al. found that cfDNA promoted multi-organ dysfunction in a sepsis model by regulating a TLR9-mediated inflammatory pathway. Functionalized and biodegradable mesoporous silica nanoparticles were synthesized using polyvinyl imine as a cfDNA scavenger. These nucleic acid-bound nanoparticles showed excellent properties and optimal *in vivo* safety for the inhibition of cfDNA-induced inflammation and multiple organ dysfunction in a model of severe sepsis (Dawulieti et al., 2020). The above studies demonstrated the feasibility of kidney-targeting cfDNA removal. Current nanomaterial sizes are in the range of 1–100 nm; the spaces between glomerular capillary endothelial cells and podocytes are 60–80 nm and 20–50 nm, respectively. Thus, a drug delivery system in the range of 70–130 nm (liposomes or nanoparticles) can reach the mesangial region without glomerular filtration and effectively remain in this region, as shown in Figure 2. In recent years, some researchers have shown that polyglycerol amine–covered nanosheets with appropriate sizes effectively reduce the serum cfDNA content and primarily accumulate in the kidney; they inhibited the formation of NETs and onset of kidney inflammation, thus alleviating AKI in mice (Wu et al., 2023).

**FIGURE 3:**
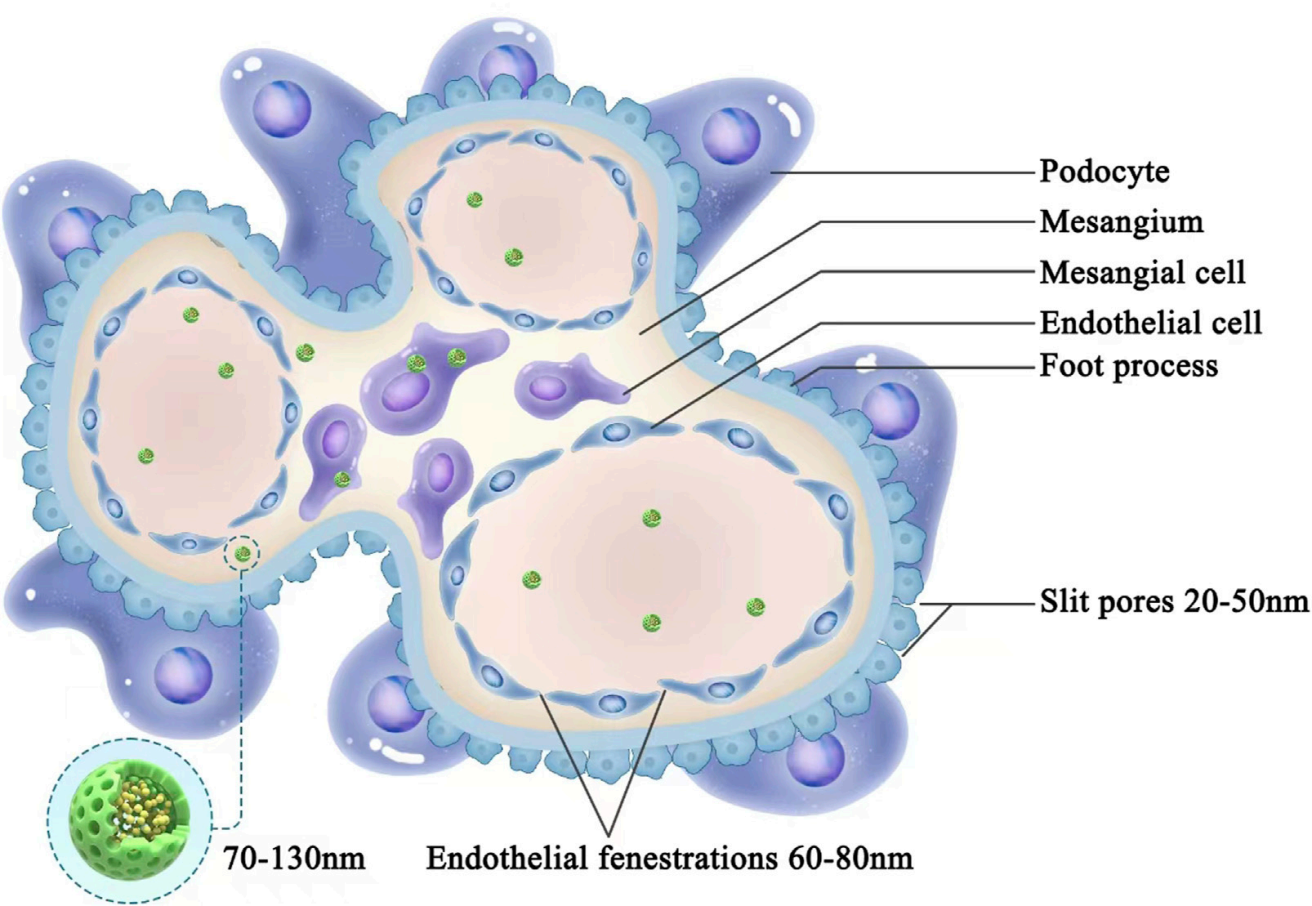
Schematic diagram of the receptor-mediated delivery of nanodrug carrier conjugates. First, nanodrug carrier conjugates cross through the endothelial fenestration. Second, nanodrug carrier conjugates are absorbed by proximal tubular epithelial cells. Third, covalent bonds between the nanodrug and carrier are cleaved upon conjugate entry into the lysosome, releasing the nanodrug from the conjugate. Fourth, nanodrugs enter the renal tubular interstitium under capillary pressure and are absorbed by proximal tubular epithelial cells.

Receptor-mediated nanodrug carrier conjugate delivery systems have been developed to target renal tubular epithelial cells and the renal interstitium; such approaches include protein-based and peptide-based carrier systems, polymer carrier systems, and antibody drug conjugates. Nanodrug carrier conjugates are designed to target renal tubular epithelial cells and the renal interstitium in the following ways. First, nanodrug carrier conjugates cross through the endothelial fenestration. Second, nanodrug carrier conjugates are absorbed by proximal tubular epithelial cells. Third, covalent bonds between the nanodrug and carrier are cleaved upon conjugate entry into the lysosome, releasing the nanodrug from the conjugate. Fourth, nanodrugs enter the renal tubular interstitium under capillary pressure and are absorbed by proximal tubular epithelial cells (Liu et al., 2019), as shown in Figure 3. Based on the current literature described above, cfDNA removal strategies can be developed to provide novel approaches for the treatment of kidney disease.

## 5 Conclusion and future perspectives

Kidney disease has serious impacts on human health worldwide. With the further understanding of the mechanisms of AKI and CKD, cfDNA, considered as a by-product of necrotic kidney cell components, promotes the progression of inflammatory response through a series of immune responses, and eventually develops into renal tubulointerstitial fibrosis and glomerulosclerosis, leading to renal failure. Therefore, cfDNA is not only a consequence, but also a cause of kidney damage. Thus, there is an urgent need to identify methods for targeted removal of renal cfDNA. The emergence of novel biological nanomaterials, combined with the unique structural features of glomeruli, has enabled the development of nanomaterial delivery systems targeting mesangial cells and tubular epithelial cells; these systems have great potential for clinical applications in the treatment of kidney disease. Although previous studies demonstrated that nanoparticles could be used for cfDNA removal, difficulties concerning efficient targeting of glomerular mesangial cells and tubular epithelial cells remain important obstacles to the development of kidney-targeting delivery systems. The main challenges for kidney-targeting cfDNA removal strategies involve the identification of a safe and efficient cfDNA removal material, as well as the establishment of efficient targeting effects in the glomerular mesangial region and renal tubular interstitium. With further understanding of kidney structure and function, and continuous optimization of vector and targeted ligands, it is promising to develop novel cfDNA removal strategies targeting the kidney to achieve precise treatment of kidney disease.
